# Rapid induction of GFP expression by the nitrate reductase promoter in the diatom *Phaeodactylum tricornutum*

**DOI:** 10.7717/peerj.2344

**Published:** 2016-08-25

**Authors:** Lili Chu, Daniela Ewe, Carolina Río Bártulos, Peter G. Kroth, Ansgar Gruber

**Affiliations:** 1 Fachbereich Biologie, Universität Konstanz, Konstanz, Germany; 2 Current affiliation: Centre Algatech, Institute of Microbiology, The Czech Academy of Science, Třeboň, Czech Republic

**Keywords:** Flow cytometry, Nitrogen source, Nitrate, Fluorescence intensity, Inducible promoter, Green fluorescent protein

## Abstract

An essential prerequisite for a controlled transgene expression is the choice of a suitable promoter. In the model diatom *Phaeodactylum tricornutum*, the most commonly used promoters for trans-gene expression are the light dependent lhcf1 promoters (derived from two endogenous genes encoding fucoxanthin chlorophyll a/c binding proteins) and the nitrate dependent nr promoter (derived from the endogenous nitrate reductase gene). In this study, we investigated the time dependent expression of the green fluorescent protein (GFP) reporter under control of the nitrate reductase promoter in independently genetically transformed *P. tricornutum* cell lines following induction of expression by change of the nitrogen source in the medium via flow cytometry, microscopy and western blotting. In all investigated cell lines, GFP fluorescence started to increase 1 h after change of the medium, the fastest increase rates were observed between 2 and 3 h. Fluorescence continued to increase slightly for up to 7 h even after transfer of the cells to ammonium medium. The subsequent decrease of GFP fluorescence was much slower than the increase, probably due to the stability of GFP. The investigation of several cell lines transformed with nr based constructs revealed that, also in the absence of nitrate, the promoter may show residual activity. Furthermore, we observed a strong variation of gene expression between independent cell lines, emphasising the importance of a thorough characterisation of genetically modified cell lines and their individual expression patterns.

## Introduction

Diatoms play a significant role in almost all aquatic ecosystems (Armbrust, 2009; Falkowski & Oliver, 2007). Besides their ecological and biogeochemical importance, diatoms also gained large interest with respect to potential biotechnological or biopharmaceutical exploitation (Hamilton et al., 2015; Hempel et al., 2011a; Hempel et al., 2011b; Hempel & Maier, 2012; Roesle et al., 2014; Vanier et al., 2015). Diatoms are interesting biotechnological targets because of their easy and cheap culturing requirements, asexual reproduction and fast growth, resulting in efficient biomass production (Bozarth, Maier & Zauner, 2009; Kilian & Kroth, 2006; Kroth, 2007a). An important prerequisite for biotechnological applications is the availability of protocols for genetic transformation of different diatom species. Meanwhile, a variety of techniques for the introduction of foreign DNA into the genomes of diatoms have been established: biolistic transformation by particle bombardment (Apt, Kroth-Pancic & Grossman, 1996; Falciatore et al., 1999), electroporation (Miyahara et al., 2013; Niu et al., 2012; Zhang & Hu, 2013) or gene transfer via conjugating bacteria (Karas et al., 2015).

To regulate transgene expression, the choice of a suitable promoter is essential. Although there is some information available regarding the functionality of heterologous regulatory DNA sequences or promoters in diatoms (Brakemann et al., 2008; Dunahay, Jarvis & Roessler, 1995; Falciatore et al., 1999; Kadono et al., 2015; Sakaue, Harada & Matsuda, 2008), all the widely utilised transformation systems for the diatoms *Phaeodactylum tricornutum* (Falciatore et al., 1999; Zaslavskaia et al., 2000), *Cylindrotheca fusiformis* (Fischer et al., 1999) and *Thalassiosira pseudonana* (Poulsen, Chesley & Kröger, 2006) employ promoters derived from the respective target organism. The availability of genome sequence data for several diatoms (Armbrust et al., 2004; Bowler et al., 2008; Lommer et al., 2012) allows the identification of putative promoter sequences that can be tested regarding their usability. Four types of endogenous promoters are currently used in the model organism *P. tricornutum*: the lhcf1 promoters, derived from two genes encoding chlorophyll *a*/*c*-binding light harvesting complex proteins (Apt, Kroth-Pancic & Grossman, 1996) (formerly known as “FCPA” and “FCPB” (Bhaya & Grossman, 1993), now referred to as “LHCF1” and “LHCF2” (Durnford, 2003)), the nitrate reductase (nr) promoter (first applied in *C. fusiformis* (Poulsen & Kröger, 2005), now also used in *T. pseudonana* (Poulsen, Chesley & Kröger, 2006) and *P. tricornutum* (Hempel et al., 2009)), the histone h4 promoter, which is used to obtain stable levels of transcription (De Riso et al., 2009) and the ef2 promoter, which has been described recently as a new tool for constitutive gene expression (Seo et al., 2015). The lhcf1 promoter are light dependent and inactive in the dark (Nymark et al., 2013). Therefore, if controlled induction of the transgene expression is required, these promoters are not suitable for photoautotrophic organisms like diatoms that do not grow in darkness. Nevertheless, lhcf promoters are currently the most widely employed promoters for trans-gene expression in *P. tricornutum*. The nr promoter activity in *C. fusiformis* is affected by the available nitrogen source (Poulsen & Kröger, 2005). Transcripts are present during cultivation with nitrate (NO_3_^−^) as nitrogen source, while no nr transcripts are detected in the presence of ammonium (NH_4_^+^) in the medium (Poulsen & Kröger, 2005). If the medium does not contain any nitrogen source, the respective transcripts accumulate without being translated (Poulsen & Kröger, 2005). Accordingly, the expression of transgenes can be controlled easily by the choice of the nitrogen source added (Poulsen & Kröger, 2005).

For in vivo localisation studies, proteins of interest are usually genetically fused to reporter genes (mostly fluorescent proteins) and then are integrated via vector DNA into the nuclear genome. In the case of *P. tricornutum*, various constructs in which the expression of marker and reporter genes is driven by the lhcf1 promoter have been reported (Apt, Kroth-Pancic & Grossman, 1996; Poulsen & Kröger, 2005; Siaut et al., 2007; Zaslavskaia et al., 2000). Since the artificially introduced vector DNA integrates randomly into the genome, each resulting transformed cell line is different, due to the random genomic position of the promoter-transgene construct, as well as possible disruption of wild type genes at the insertion site. Accordingly, the intensity of transgene expression can vary in the different transformed cell lines (Apt, Kroth-Pancic & Grossman, 1996; Poulsen & Kröger, 2005; Zaslavskaia et al., 2000). In this study, we chose several nr-GFP expressing cell lines of *P. tricornutum* in order to compare the promoter’s efficiency among different cell lines. We induced and inactivated the nr promoter by changing the nitrogen source in the medium, observed the development of the gene product green fluorescent protein (GFP) by flow cytometric analyses in comparison to a cell line expressing GFP under control of the lhcf1 promoter. Hereby, it became obvious that the cell lines differ from each other regarding the quantity of the expressed target protein. Interestingly, the nr promoter cannot be fully down regulated by the choice of the nitrogen source.

## Materials and Methods

### Culture conditions

*Phaeodactylum tricornutum* Bohlin (University of Texas Culture Collection, Austin, strain UTEX646) (denoted “Pt4” by Martino et al. (2007)) was grown in artificial half-concentrated seawater (16.6 g L^−1^, Tropic Marin, Dr. Biener GmbH, Wartenberg/Angersbach, Germany) enriched with f/2 nutrition as described in Guillard (1975) and additionally buffered with Tris pH 8 (2 mM, half of the concentration suggested for “f-1” by Guillard & Ryther (1962)). Growth media were supplemented with different nitrogen compounds: NaNO_3_ (0.882 mM, medium A, corresponding to the original f/2 nutrition) or NH_4_Cl (0.882 mM, medium B). Cells were grown at 18–20 °C and 75 μmol photons m^−2^ s^−1^, either in liquid culture in Erlenmeyer flasks on a horizontal shaker (120 rpm), or on f/2 agar plates with solid media containing 1.2% (w/v) Bacto Agar (Becton, Dickinson and Company, Le Pont de Claix, France).

### Transformation vector and plasmid constructions

Standard cloning procedures were performed for plasmid construction (Sambrook, Fritsch & Maniatis, 1989). The *P. tricornutum* transformation vector pPha-T1 (GenBank accession number AF219942.1) (Zaslavskaia et al., 2000) was utilised for cloning. The EcoRV restriction site was used to insert the gene for enhanced GFP (eGFP), giving rise to the plasmid pPha-T1-GFP (Gruber et al., 2007). A second plasmid was constructed based on the *P. tricornutum* transformation vector pPha-NR (GenBank accession number JN180663.1; Stork et al. (2012)). The eGFP gene (Clontech, Palo Alto, CA) was cloned downstream of the nr promoter sequence using the EcoRV restriction site.

### Biolistic transformation

Cells were transformed using the Biolistic PDS-1000/He Particle Delivery System (Bio-Rad, Hercules, California, USA) fitted with 1,350 psi rupture discs as described in Kroth (2007b). After transformation, cells were allowed to recover for 24 h before being plated onto f/2 medium containing 75 μg/mL zeocin (Invitrogen, Molecular Probes, Eugene, USA) for selection. The plates were incubated at 22 °C under constant illumination (75 μmol photons m^−2^ s^−1^). One of the resulting transformed cell lines of the transformation with the plasmid pPha-T1-GFP and six of the transformed cell lines expressing GFP under control of the nitrate reductase promoter were chosen based on GFP expression screened by flow cytometry or fluorescence microscopy (see below).

### Determination of cell density

The cell concentrations were determined using a Multisizer 3 (Beckman Coulter, Brea, CA, USA) as described in Rottberger, Gruber & Kroth (2013), over a time period of 11 days. Samples were taken once per day.

### Western blot analyses and SDS-PAGE

For western blot analyses, selected *P. tricornutum* cell lines were grown at 18 °C and 75 μmol photons m^−2^ s^−1^ in continuous light. Wild type cells, the lhcf1-GFP and nr-GFP transformant cell lines were grown in medium A containing NaNO_3_, and in medium B containing NH_4_Cl. Cells were harvested during exponential phase by centrifugation (3,000 g, 10 min, 4 °C) and the pellets were resuspended in 1 mL lysis buffer containing protease inhibitor “complete EDTA-free” (Roche, Mannheim, Germany), 50 mM Tris HCl pH 8, 1 mM EDTA, and 1% (w/v) SDS. A mixture of glass beads (0.1–1 mM diameter) was added and cells were homogenised in a MP FastPrep-24™ 5G (MP Biomedicals, Santa Ana, CA, USA) at a speed of 6 m/s for four times 20 s, with 1 min breaks on ice in between the homogenisation pulses. Samples were centrifuged again (20,000 g, 30 min, 4 °C), and supernatant was transferred into a new tube and further used for SDS-PAGE. Total protein concentration was determined using the 660 nm Pierce Protein Assay (Thermo Scientific, Rockford, USA) and the spectrophotometer Ultrospec™ 8000 (GE Healthcare, Little Chalfont, UK). Each well of the gels was loaded with 3 μg of each protein extract. Proteins were separated by SDS-PAGE in 12% acrylamide gel (Laemmli, 1970) and transferred electrophoretically onto a nitrocellulose membrane (Amersham Protran 0.1 NC, GE Healthcare), using Chromatography paper (Whatman™ 3MM Chr, GE Healthcare) and a Trans-Blot Turbo (Bio-Rad) at 1.3 A and 25 V for 12 min. Page Ruler Prestained Protein Ladder (Thermo Scientific, Schwerte, Germany), primary antibody α-GFP (catalog number A-6455, Invitrogen), diluted 1:10,000, and secondary antibody α-Rabbit IgG (catalog number A0545, Sigma Aldrich, Munich, Germany), diluted 1:20,000, were utilised. Roti®-Block, Roti®-Lumin plus (Carl Roth GmbH & Co. KG, Karlsruhe, Germany) and InstantBlue™ (Expedeon, San Diego, CA, USA) were applied as described in their manuals. Immunodetection was performed with the Odyssey® Fc Imaging System (LI-COR Biosciences, Lincoln, NE, USA).

### Induction of nitrate reductase promoter

Before fluorescence was measured, the cell lines were kept for several days in liquid ammonium-containing medium in multi-well plates under continuous light illumination at 75 μmol photons m^−2^ s^−1^ and 18 °C to make sure that the nr promoter was switched off and that no GFP was visible in the nr-GFP transformed cell lines. For the measurements, cultures were inoculated in fresh ammonium-containing medium and were harvested after three days during exponential phase by centrifugation (3,000 g, 10 min). The fluorescence measurements (BD FACSCalibur, BD Biosciences, CA, USA) were started after the cells were washed once by resuspension in fresh nitrate medium and another centrifugation step (time point 0 h). The samples were taken after different time intervals to observe changes in fluorescence intensity. After 24 h in nitrate-containing medium, the nr promoter was switched off again by harvesting, washing and transferring the cells back into ammonium-medium. The cells were observed for another 10 days (264 h in total) with samples taken at the indicated intervals. Microscopic analyses were performed in parallel to verify potential GFP fluorescence.

### Flow cytometry

Flow cytometric analyses were performed using the flow cytometer BD FACSCalibur (BD Biosciences) and the Software BD CellQuestPro (BD Biosciences). For the detection of GFP, we used the 488 nm laser for excitation and the FL1 detector with a 530/30 BP filter for detection. For the detection of red fluorescence, we utilised in parallel the FL3 detector with a 650 LP filter. The emission intensities of 100,000 cells per sample (triggered by side scatter) were collected and ungated median fluorescence intensities of each population were collected for the subsequent analyses. Dot plots and histograms were created and analysed using the Single Cell Analysis Software FlowJo (Tree Star, Inc., Ashland, OR). The raw data exported from the flow cytometer BD FACSCalibur and the FlowJo workspace applied for data analyses and figure preparation are contained in Data S1.

Wild type cell cultures were used as negative control and an lhcf1-GFP transformed cell line as reference for a GFP-expressing cell line under the control of a nitrate-independent promoter.

### Fluorescence microscopy

Cellular expression of GFP fusion proteins was analysed with an epifluorescence microscope Olympus BX51 (Olympus Europe, Hamburg, Germany), a Zeiss AxioCam MRm digital camera (Carl Zeiss, Oberkochen, Germany) and an Olympus PLN 40× objective (Olympus Europe, Hamburg, Germany). Image processing was conducted using the Software AxioVision Rel. 4.7 (Carl Zeiss, Oberkochen, Germany).

## Results

We generated genetically transformed cell lines of *Phaeodactylum tricornutum* expressing GFP, under the control of the nr promoter. We selected six nr strains with varying intensities of GFP fluorescence using fluorescence microscopy. Additionally, as a reference for a nitrate-independent promoter, we chose a GFP-expressing cell line under control of the lhcf1 promoter (Table 1).

**Table 1:** Wild type and GFP expressing cell lines of *P. tricornutum* used for time-dependent GFP-fluorescence measurements by flow cytometry and fluorescence microscopy. Protein IDs refer to wild type strain CCMP632 (denoted “Pt1” by Martino et al. (2007)), which was sequenced by the U.S. Department of Energy Joint Genome Institute (http://genome.jgi.doe.gov/Phatr2/Phatr2.home.html) (Bowler et al., 2008).

Cell line name	Description
Wt	Wild type *Phaeodactylum tricornutum* Bohlin (University of Texas Culture Collection, Austin, strain UTEX646) (denoted “Pt4” by Martino et al. (2007))
lhcf1-GFP	*P. triconutum* UTEX646, genetically transformed with the pPha-T1-GFP plasmid (Gruber et al., 2007), derived from pPha-T1 (GenBank AF219942.1, Zaslavskaia et al. (2000)), which contains the 442 bp 5′-flanking region of the *P. triconutum* lhcf1 gene (equivalent protein in Pt1, ID 18049) (Apt, Kroth-Pancic & Grossman, 1996) as promoter
nr-GFP_3	*P. triconutum* UTEX646, genetically transformed cell lines expressing the eGFP gene with a construct derived from the pPha-NR vector (GenBank JN180663.1, Stork et al. (2012)), which contains the 422 bp 5′-flanking region of the *P. triconutum* nr gene (equivalent protein in Pt1, ID 54983) (Hempel et al., 2009) as promoter (see text for details)
nr-GFP_4	
nr-GFP_5	
nr-GFP_6	
nr-GFP_9	
nr-GFP_10	

**Figure 1:**
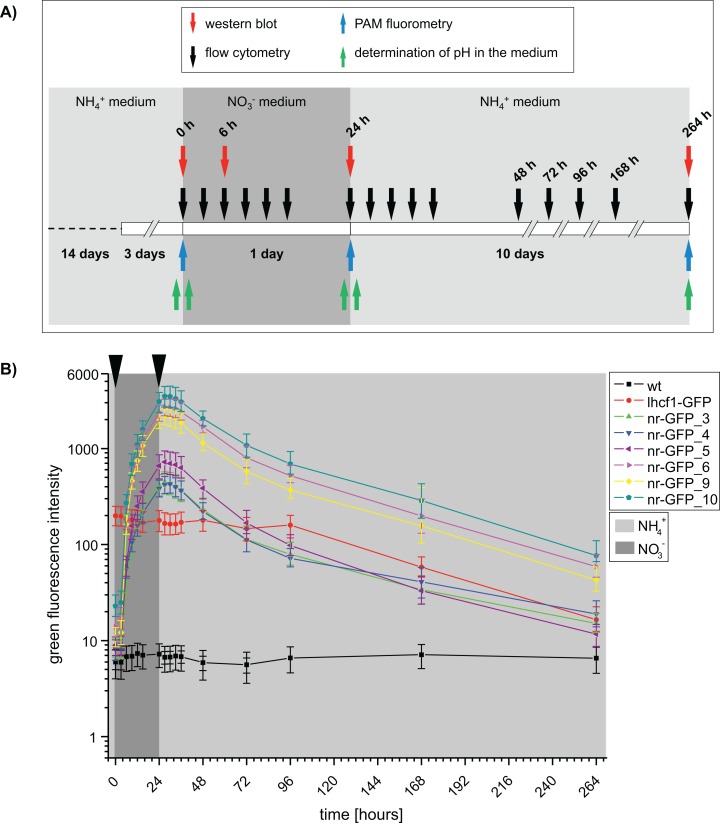
(A) Time scale of the experimental setup. Arrows indicate the time points of sampling. Western blots and flow cytometry was performed using *P. tricornutum* wild type and GFP-expressing cell lines. PAM (Pulse amplitude modulation) fluorometry of cell cultures and pH of the media was determined using wild type cell lines. h = hours. (B) Median green fluorescence intensities determined for *P. tricornutum* wild type and GFP-expressing cell lines. Intensities were determined using the flow cytometer BD FACSCalibur at indicated time intervals. Arrows indicate washing steps and medium change. Error bars represent the interquartile range (IQR).

To determine the velocity of GFP synthesis induced by a change of nitrogen source in the medium, we set up a time dependent experiment with sampling at different time points (Fig. 1). We determined the green and red fluorescence intensities with the flow cytometer BD FACSCalibur in regular time intervals after induction of the nr promoter by transferring the cells from ammonium- to nitrate-medium (Fig. S1; Tables S1 and S2).

Dot plots of green versus red channel signals show that the signal level in the red channel is similar between GFP expressing cell lines and wild type cell lines. This means that the chosen filter sets efficiently separated the green GFP fluorescence from the red chlorophyll autofluorescence and that the signal in the green channel did not result from spillover of the chlorophyll autofluorescence (Fig. S2; Table S2).

Furthermore, we checked for possible physiological effects of the medium changes on *P. tricornutum* wild type cells by PAM (pulse-amplitude modulation) fluorometry and by measuring the pH of the medium (Tables S3 and S4). The pH of the medium changed only slightly and remained around pH 8 throughout the whole experiment (Table S3). Ratios of variable fluorescence to maximal fluorescence (Fv/Fm) changed slightly between nitrate and ammonium media, while non-photochemical quenching (NPQ) stayed constant (Table S4). Growth measurements confirmed that cell growth was similar between all investigated cell lines and independent from the nitrogen source present in the media. All cultures entered the stationary growth phase after ∼120 h (Fig. S3). The red autofluorescence intensities remained on a similar level in all of the cell lines until ∼168 h of the experiment and dropped slightly towards the end of the experiment (Table S2), indicating changes in the pigment content while the cells enter the stationary growth phase.

At the beginning of the induction experiment, the green fluorescence signals of all genetically transformed cell lines were higher than in the wild type cells (Fig. S1; Table S1A). In case of the nr-GFP cell lines, the green fluorescence signal indicated the presence of a certain GFP-level in the cells even during cultivation in ammonium-medium (Fig. 1; Table S1), which could also be shown for several cell lines via western blots (Fig. S4). In case of the lhcf1-GFP reference cell line, the level of green fluorescence did not change throughout the induction experiment, with the exception of a decrease in fluorescence first measured after 96 h of the experiment (Fig. 1; Table S1B). Simultaneously decreasing autofluorescence signals indicated that this might be related to ageing of the culture (Table S2B).

Upon the start of the experiment, after ammonium acclimated cells had been transferred into nitrate-medium, green fluorescence increased in all nr-GFP cell lines throughout the cultivation time, with the highest rates of increase between 3 and 6 h after the change of the medium (Table S1A). The GFP signal was not detectable microscopically until about 6 h after the medium change. Throughout the rest of the experiment, microscopic GFP fluorescence detection subjectively did not change, although the flow cytometer indicated an increase of GFP fluorescence intensity of up to twelve-fold between 6 and 24 h after induction (Fig. S5; Table S1A).

After all cell lines had been incubated for 24 h in nitrate-medium, they were transferred back into ammonium-containing medium and subsequent measurements showed decreasing green fluorescence in nearly all nr-GFP cell lines 3–6 h after the medium change (27–30 h, Table S1B). The rate of decrease of green fluorescence was much slower than the rate of increase. Even after 264 h, the green fluorescence intensities in the nr-GFP cell lines were higher than at the beginning of the experiment. Due to the age of the cultures, the measurements after 168 and 264 h generally show low fluorescence in both the green and red channel (including the positive control lhcf1-GFP, Tables S1B and S2B). Since the chlorophyll autofluorescence of the cells was also lower than at the beginning of the measurements, the low green fluorescence signal does not specifically imply a decrease in the steady state levels of fluorescing GFP, but could also reflect the state of the cells in late stationary growth phase (Tables S1 and S2).

**Figure 2:**
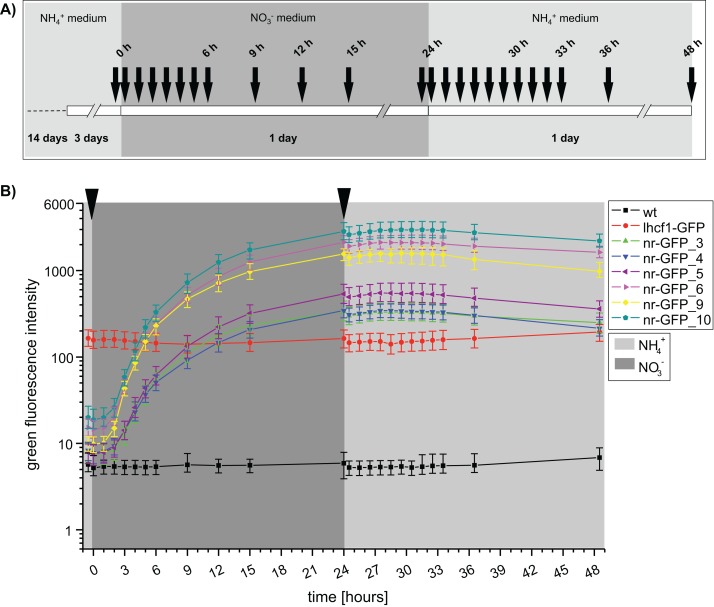
(A) Time scale of flow cytometry sampling using *P. tricornutum* wild type cell line and GFP-expressing cell lines, h = hours. (B) Median green fluorescence intensities determined for *P. tricornutum* wild type and GFP-expressing cell lines. Intensities were determined using the flow cytometer BD FACSCalibur at indicated time intervals. Arrows indicate washing steps and medium change. Error bars represent the interquartile range (IQR).

An independent repetition of the fluorescence measurements with a higher temporal resolution (Fig. 2A), confirmed that GFP fluorescence was not immediately decreasing after transferring the nr cell lines back into ammonium-medium (Fig. 2B). To the contrary, we could still observe a slight increase of green fluorescence for the next 3 h (27 h), albeit the rate of increase was immediately lower after the medium change. A first decrease of GFP intensity could be observed after 3 h (27 h) in the cell lines nr-GFP_5, _6 and _9. However, from this time point, the intensities remained constant in all of the nr cell lines before finally decreasing after 7 h (31 h).

In order to assess the range of fluorescence intensities obtained in cell lines resulting from independent genetic transformation events with identical constructs, we repeated transformation of wild type *P. tricornutum* with the lhcf1-GFP construct and screened all resulting zeocin resistant colonies for GFP expression. The genetic transformation resulted in 45 cell lines, most of these showed detectable GFP fluorescence (Fig. S6). The majority of the cell lines showed lower relative GFP fluorescence values than the nr-GFP cell lines (compare to Figs. 1 and 2).

## Discussion

Transgene expression in the diatom *Phaeodactylum tricornutum* in current biotechnological approaches is usually based on the lhcf1 or nr promoters (Apt, Kroth-Pancic & Grossman, 1996; Hempel et al., 2009; Zaslavskaia et al., 2000). In wild type cells, the lhcf1 promoter drives the expression of a light harvesting complex protein, a member of a large multigene family (Durnford, 2003; Sturm et al., 2013). Light dependency of transcription was confirmed in *P. tricornutum* for LHCF2 (FCPB) (Lepetit et al., 2013; Russo et al., 2015; Sturm et al., 2013) and further studies on gene expression showed similar patterns for LHCF2 (Protein ID 25172) and LHCF1 (Protein ID 18049) (Maheswari et al., 2010; Maheswari et al., 2009; Nymark et al., 2009; Nymark et al., 2013; Valle et al., 2014). In our study the lhcf1-GFP *P. tricornutum* strain served as a nitrate-independent reference strain. Using flow cytometry, GFP as reporter gene could be traced accurately, this approach consequently enables the characterisation of additional promoters for basic or applied research.

The nr promoter activity is inducible by a change of the nitrogen source in the media (Poulsen & Kröger, 2005). However, we also observed a slight presence of GFP in all of the nr-GFP cell lines before the promoter was induced by nitrate. This implies that the nr promoter is ‘leaky’ in the presence of ammonium and a certain background level of the gene product is maintained.

Our data indicates that the GFP fluorescence in most of the investigated nr-GFP cell lines is stronger compared to the lhcf1-GFP cell line (compare Figs. 1, 2 and S6). This strength of the nr promoter might be useful for the overexpression of trans-genes, when high protein levels are required. However, excessive protein expression sometimes comes with the risk of disturbing cellular homeostasis (in biotechnological applications) or the experiments themselves (for instance in protein localisation studies). In such situations, selection of transformed cell lines with low expression levels or careful adjustments of the ammonium:nitrate ratio in the media would be required.

During cultivation in nitrate free ammonium containing medium, the nr promoter was reported to be inactive (Poulsen & Kröger, 2005). The details of the process of inactivation are not entirely clear. It is suggested that ammonium addition inhibits the uptake of nitrate by the cells (Cresswell & Syrett, 1979). Our data indicate, that inactivation of the promoter is a slow process, since we could observe a first decrease of GFP intensity in several nr cell lines only 3 h after change of the medium. The residual increase of GFP fluorescence within the first 3 h could be caused by translation of remaining GFP transcripts in the cell. Between 3 and 7 h, the GFP amount appears to stay on a static level, indicating that no additional GFP molecules are synthesised. The subsequent decrease of GFP may either occur by dilution of the GFP amount per cell during every cell division, or by degradation of the GFP.

Since the cells were kept in ammonium-medium for several days, they already reached the stationary growth phase. It was demonstrated that the addition of any particular nutrient alone does not lead to a positive growth response (Rottberger et al., 2013), however, a combination of added nutrients might be sufficient to trigger cell division. Hence, if depletion of GFP in a culture is required, we suggest transferring the cells into fresh media containing ammonium. Alternatively, the use of a destabilised green fluorescent protein might be helpful (see Dantuma et al., 2000; Houser et al., 2012; Kitsera, Khobta & Epe, 2007; Li et al., 1998), especially to track rapid changes in gene expression and protein turnover.

Complete nitrogen deprivation in *P. tricornutum* reportedly results in a decrease of photosynthetic capacity and chlorophyll content, and in a simultaneous accumulation of neutral lipids (Alipanah et al., 2015; Valenzuela et al., 2012; Yu et al., 2009). Also in our experiments the intensity of the red plastid autofluorescence decreased not before 96 h in ammonium-medium (Table S2B). We also observed accumulation of lipid droplets within the cells towards the end of the cultivation interval (Fig. S5), which is a typical phenotype for nitrogen starvation (Ge et al., 2014; Yang et al., 2013; Levitan et al., 2014).

The characterisation of several nr-GFP and lhcf1-GFP cell lines revealed that transgene expression does not necessarily lead to the same expression pattern among the cell lines, although they were transformed with identical DNA. In addition to external factors, like medium, light and growth phase, each transgene promoter activity also depends on the position of the vector DNA insertion within the host genome. It has been shown for cells transformed by particle bombardment that vector DNA integration varies in the number and position of insertions (Zaslavskaia et al., 2000) leading to so far unpredictable variations in transgene expression (Zaslavskaia et al., 2000, this study). In the future, this might be overcome by using targeted gene insertion systems like TALEN (transcription activator-like effector nucleases, Daboussi et al., 2014) CRISPR/Cas9 (clustered regularly interspaced short palindromic repeats, Nymark et al., 2016), or by transformation systems which lead to stable episomal replication of the plasmids instead of integration of trans-genes into the nuclear genome (Karas et al., 2015).

## Supplemental Information

10.7717/peerj.2344/supp-1 Supplemental Information 1 Histograms showing green fluorescence development of *P. tricornutum* wild type (blue) and genetically transformed cell lines (red) after transfer into nitrate-medium. The green fluorescence intensity has been plotted in log-scale (X-axis) versus cell counts detected by scattered light (Y-axis). Numbers indicate median green fluorescence intensity of 100,000 cells of each transformed cell line (red) and of the wild type cell line (blue). h = hours. * = dot plot shown in Fig. S2.

10.7717/peerj.2344/supp-2 Supplemental Information 2 Dot plots (compare Fig. S1) of *P. tricornutum* cell lines 0 hours (0 h) and 24 hours (24 h) after the cells were transferred into NO_3_^−^-medium. Green fluorescence (X-axis) is plotted versus autofluorescence of chlorophyll (Y-axis). Data of each population (1,00,000 counts) of transformed cell lines are shown in red for transformed cell lines and in blue for wild type cell line.

10.7717/peerj.2344/supp-3 Supplemental Information 3 Cell numbers determined for *P. tricornutum* wild type and transformed cell lines. All cell lines were kept in NH_4_^+^-medium (light grey) before transferred into NO_3_^−^-medium (dark grey) for 24 h and subsequent transfer back into NH_4_^+^-medium. Arrows indicate washing steps and medium change.

10.7717/peerj.2344/supp-4 Supplemental Information 4 Analysis of GFP protein expression by western blot. Total protein extract (3 μg) of *P. tricornutum* wild type (wt) and transformant cell lines (lhcf1-GFP; nr-GFP_3, _4, _5, _6, _9, _10) and GFP-antibody were used for detection. Left: western blot; right: loading control. GFP-expression by nr promoter is stronger compared to the lhcf1 promoter. GFP (27 kDa) even appears in the coomassie stained loading control of nr-GFP_6, _9 and _10.

10.7717/peerj.2344/supp-5 Supplemental Information 5 Fluorescence micrographs of the *P. tricornutum* transformant cell line nr-GFP_3. Images were taken 0 hours (0 h), 6 hours (6 h), 24 hours (24 h) after transfer from NH_4_^+^-medium into NO_3_^−^-medium, and after back-transfer into NH_4_^+^-medium (264 h). Arrows indicate lipid droplets. GFP fluorescence is shown in green, autofluorescence of chlorophyll in red, and Nomarski differential interference contrast (DIC) in grey scale. Scale bars: 5 μm.

10.7717/peerj.2344/supp-6 Supplemental Information 6 Box plot of median green fluorescence intensities. The intensities were determined for a *P. tricornutum* wild type cell line (red) and 45 lhcf1-GFP transformed cell lines (green). Whiskers represent min-max-range.

10.7717/peerj.2344/supp-7 Supplemental Information 7 Green fluorescence intensity data of *P. tricornutum* wild type (wt) and GFP-expressing cell lines (lhcf1-GFP; nr-GFP_3, _4, _5; _6; _9; _10). Given data show ungated 25% quartile (25%), Median, 75% quartile (75%) and interquartile range (IQR) of the green fluorescence intensities determined for each population (1,00,000 counts). A = medium A (nitrate); B = medium B (ammonium); h = hours.

10.7717/peerj.2344/supp-8 Supplemental Information 8 Red fluorescence intensity data of *P. tricornutum* wild type (wt) and GFP-expressing cell lines (lhcf1-GFP; nr-GFP_3, _4, _5; _6; _9; _10). Given data show ungated 25% quartile (25%), Median, 75% quartile (75%) and interquartile range (IQR) of the green fluorescence intensities determined for each population (1,00,000 counts). A = medium A (nitrate); B = medium B (ammonium); h = hours.

10.7717/peerj.2344/supp-9 Supplemental Information 9 pH-determination of the NH_4_^+^ (ammonium) and NO_3_^−^ (nitrate) media for the cultivation of *P. tricornutum* wild type cell lines in triplicates. riplicates. The cells were kept in NH_4_^+^-medium for 2 weeks before inoculation into fresh NH_4_^+^-medium. After 3 days cells were transferred into NO_3_^−^-medium for 24 h and subsequent back-transfer into NH_4_^+^-medium and cultivation for another 10 days (264 h). Cell were removed by filtration prior to pH-measurements.

10.7717/peerj.2344/supp-10 Supplemental Information 10 Pulse Amplitude Modulated (PAM) fluorometry. Wild type cells were cultured in triplicates and washed in the same procedure as the one used for the GFP fluorescence measurements. After washing, cells were resuspended in NH_4_^+^ or NO_3_^−^ media at a cell density of 2 × 10^6^cells/ml. PAM measurements were performed in Plastibrand (BRAND GmbH & Co. KG, Wertheim Germany) PMMA cuvettes with an AquaPen AP-C 100 (Photon Systems Instruments, spol. s r.o., Brno, Czech Republic) using the NPQ 2 protocol with actinic light at 700 μmol photons m^−1^ s^−1^ and saturating flashes at 2,100 μmol photons m^−1^ s^−1^, the blue measuring light was adjusted to 0.0099 μmol photons m^−1^ s^−1^.

10.7717/peerj.2344/supp-11 Supplemental Information 11 Raw data exported from the flow cytometer BD FACSCalibur applied for data analyses and preparation for Fig. 1 and Tables S1 and S2 for the time period of 0–6 h.

10.7717/peerj.2344/supp-12 Supplemental Information 12 Raw data exported from the flow cytometer BD FACSCalibur applied for data analyses and preparation for Fig. 1 and Tables S1 and S2 for the time period of 6–15 h.

10.7717/peerj.2344/supp-13 Supplemental Information 13 Raw data exported from the flow cytometer BD FACSCalibur applied for data analyses and preparation for Fig. 1 and Tables S1 and S2 for the time period of 24–36 h. Raw data exported from the flow cytometer BD FACSCalibur used for data analyses and figure preparatio.

10.7717/peerj.2344/supp-14 Supplemental Information 14 Raw data exported from the flow cytometer BD FACSCalibur applied for data analyses and preparation for Fig. 1 and Tables S1 and S2 for the time point of 48 h.

10.7717/peerj.2344/supp-15 Supplemental Information 15 Raw data exported from the flow cytometer BD FACSCalibur applied for data analyses and preparation for Fig. 1 and Tables S1 and S2 for the time point of 72 h.

10.7717/peerj.2344/supp-16 Supplemental Information 16 Raw data exported from the flow cytometer BD FACSCalibur applied for data analyses and preparation for Fig. 1 and Tables S1 and S2 for the time point of 96 h.

10.7717/peerj.2344/supp-17 Supplemental Information 17 Raw data exported from the flow cytometer BD FACSCalibur applied for data analyses and preparation for Fig. 1 and Tables S1 and S2 for the time point of 168 h.

10.7717/peerj.2344/supp-18 Supplemental Information 18 Raw data exported from the flow cytometer BD FACSCalibur applied for data analyses and preparation for Fig. 1 and Tables S1 and S2 for the time point of 264 h.

10.7717/peerj.2344/supp-19 Supplemental Information 19 Raw data exported from the flow cytometer BD FACSCalibur applied for data analyses and preparation for Fig. 1 and Tables S1 and S2 by using FlowJo.

10.7717/peerj.2344/supp-20 Supplemental Information 20 Raw data exported from the flow cytometer BD FACSCalibur applied for data analyses and preparation for the detailed investigation shown Fig. 2 for the time period of 0–2 h.

10.7717/peerj.2344/supp-21 Supplemental Information 21 Raw data exported from the flow cytometer BD FACSCalibur applied for data analyses and preparation for the detailed investigation shown Fig. 2 for the time period of 2–6 h.

10.7717/peerj.2344/supp-22 Supplemental Information 22 Raw data exported from the flow cytometer BD FACSCalibur applied for data analyses and preparation for the detailed investigation shown Fig. 2 for the time period of 6–15 h.

10.7717/peerj.2344/supp-23 Supplemental Information 23 Raw data exported from the flow cytometer BD FACSCalibur applied for data analyses and preparation for the detailed investigation shown Fig. 2 for the time period of 24–26 h.

10.7717/peerj.2344/supp-24 Supplemental Information 24 Raw data exported from the flow cytometer BD FACSCalibur applied for data analyses and preparation for the detailed investigation shown Fig. 2 for the time period of 27–30 h.

10.7717/peerj.2344/supp-25 Supplemental Information 25 Raw data exported from the flow cytometer BD FACSCalibur applied for data analyses and preparation for the detailed investigation shown Fig. 2 for the time period of 31–36 h.

10.7717/peerj.2344/supp-26 Supplemental Information 26 Raw data exported from the flow cytometer BD FACSCalibur applied for data analyses and preparation for the detailed investigation shown Fig. 2 for the time point of 48 h.

10.7717/peerj.2344/supp-27 Supplemental Information 27 Raw data exported from the flow cytometer BD FACSCalibur applied for data analyses and preparation for Fig. 1 and Tables S1 and S2 for the time period of 0–15 h by using FlowJo.

10.7717/peerj.2344/supp-28 Supplemental Information 28 Raw data exported from the flow cytometer BD FACSCalibur applied for data analyses and preparation for Fig. 1 and Tables S1 and S2 for the time period of 24–36 h by using FlowJo.

10.7717/peerj.2344/supp-29 Supplemental Information 29 Raw data exported from the flow cytometer BD FACSCalibur applied for data analyses and preparation for Fig. 1 and Tables S1 and S2 for the time point of 48 h by using FlowJo.

## Additional Information and Declarations

### Data Deposition

The following information was supplied regarding data availability: The raw data has been supplied as Supplemental Dataset Files.
