# Supplementary figures and images for: Rapid induction of GFP expression by the nitrate reductase promoter in the diatom Phaeodactylum tricornutum
Source: PeerJ. 2016 Aug 25;4:e2344. doi: 10.7717/peerj.2344 (PMC5012323; doi:10.7717/peerj.2344)

0 h

6 h

24 h

264 h

 $\text{NO}_3^-$  $\text{NH}_4^+$ 

lhcf1-GFP

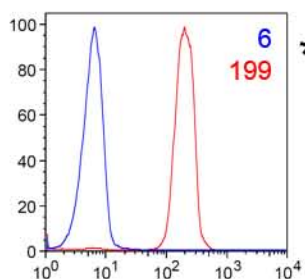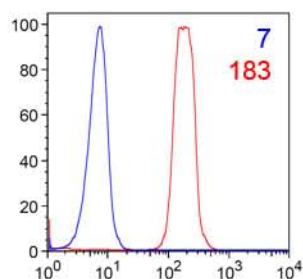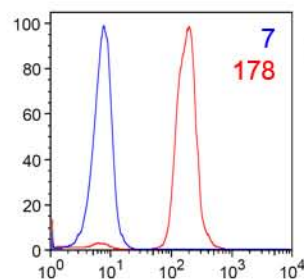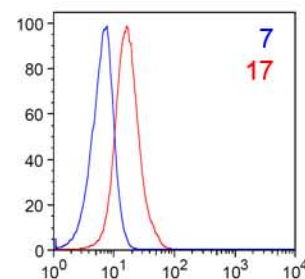

nr-GFP\_3

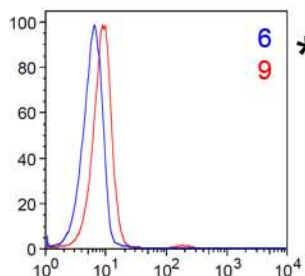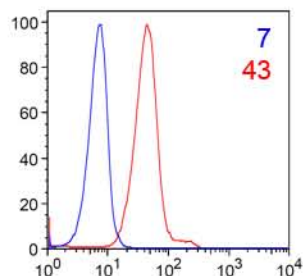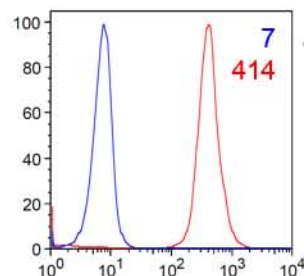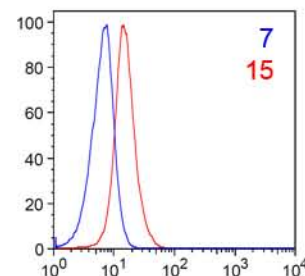

nr-GFP\_4

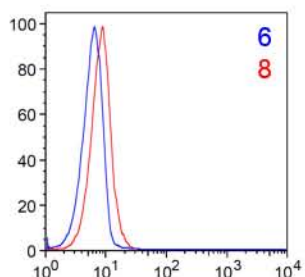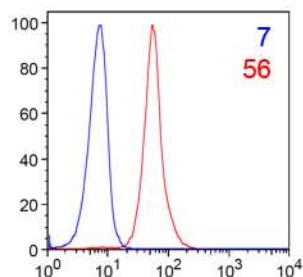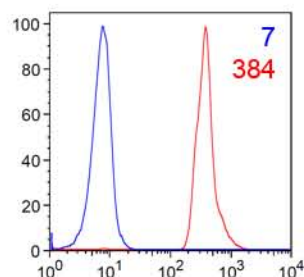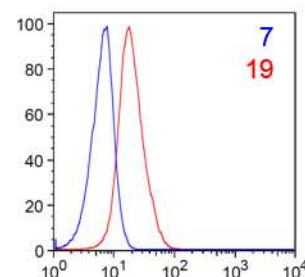

nr-GFP\_5

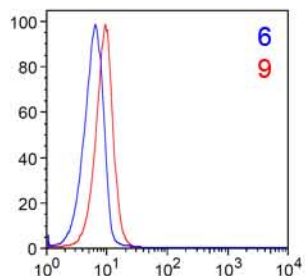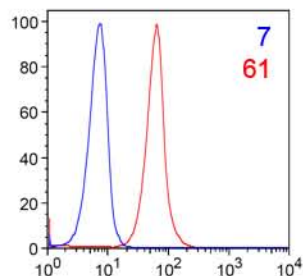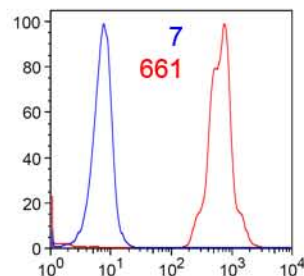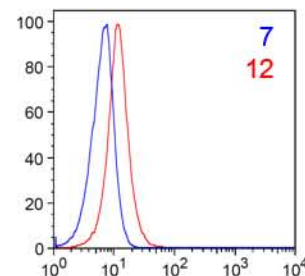

nr-GFP\_6

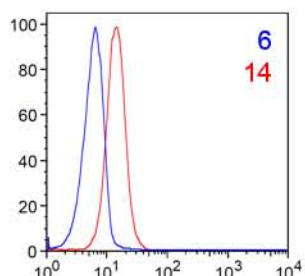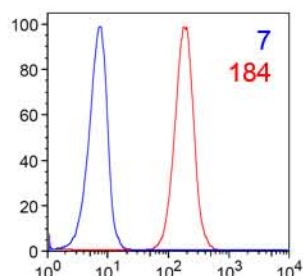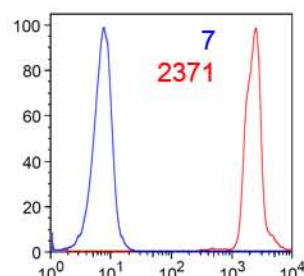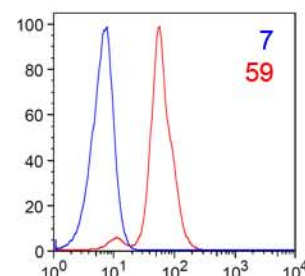

nr-GFP\_9

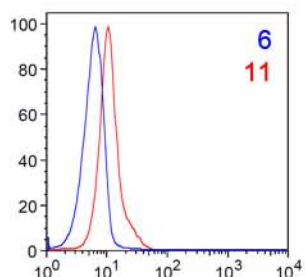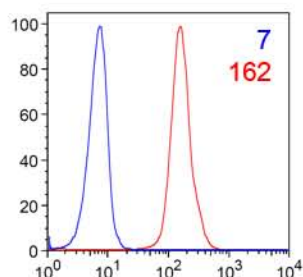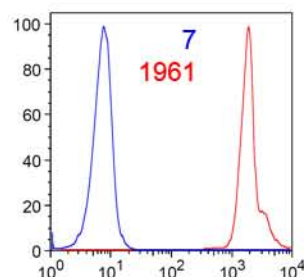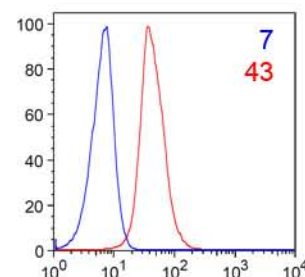

nr-GFP\_10

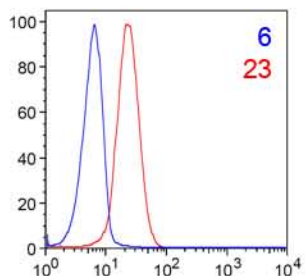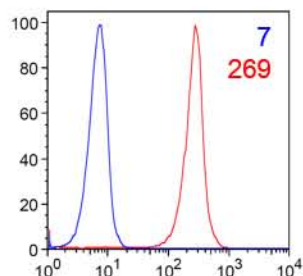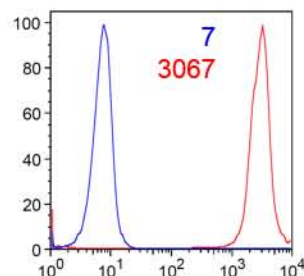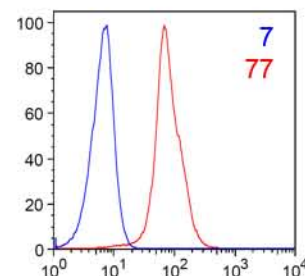

Supplement: Supplemental Information 1 — The green fluorescence intensity has been plotted in log-scale (X-axis) versus cell counts detected by scattered light (Y-axis). Numbers indicate median green fluorescence intensity of 100,000 cells of each transformed cell line (red) and of the wild type cell line (blue). h = hours. * = dot plot shown in Fig. S2. [file peerj-04-2344-s001.pdf]

0 h

24 h

lhcf1-GFP

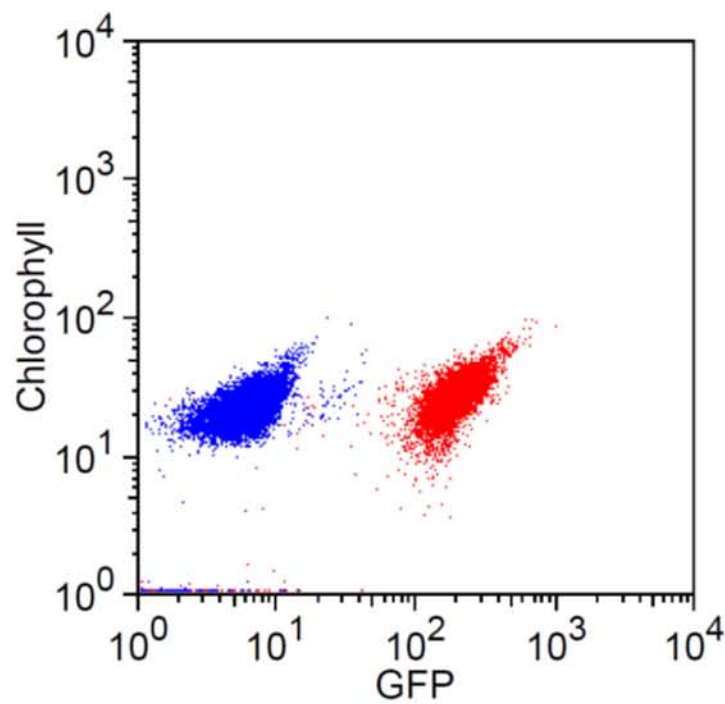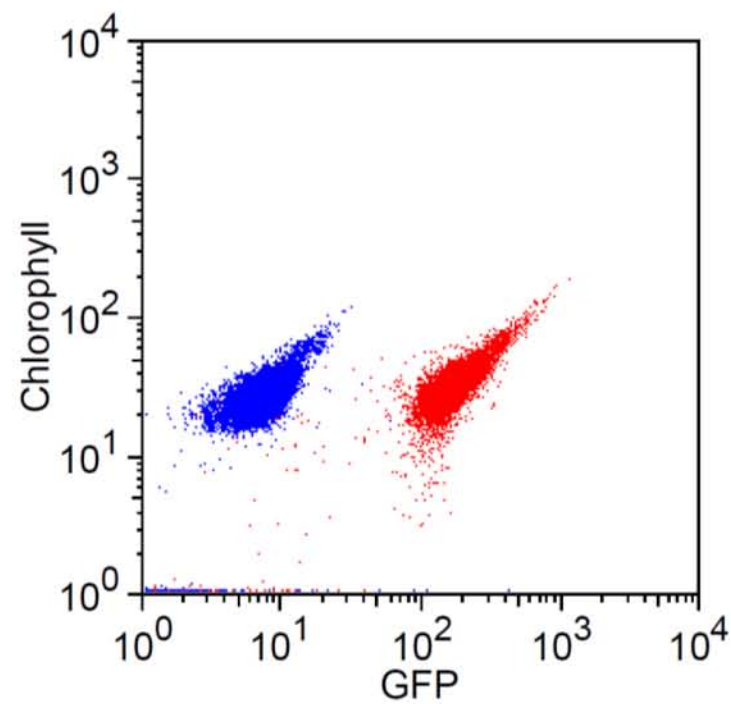

nr-GFP\_3

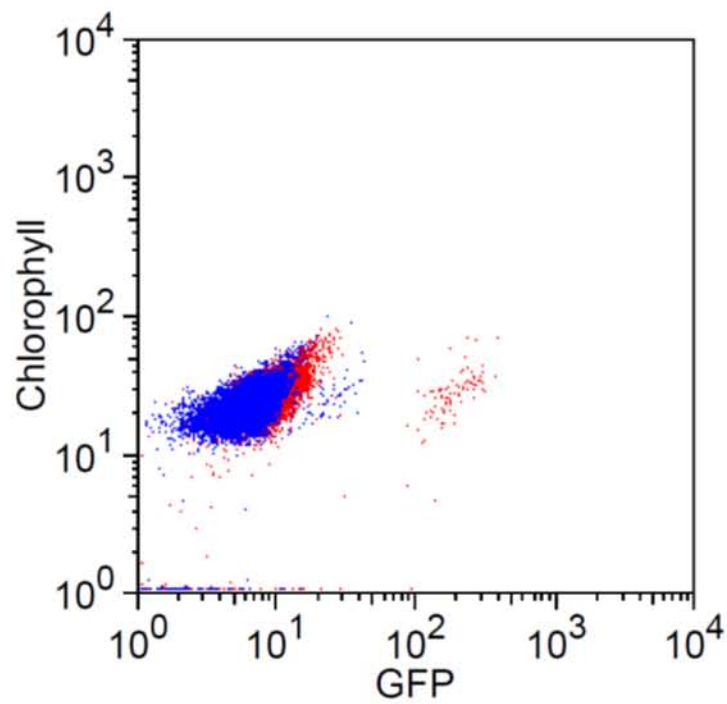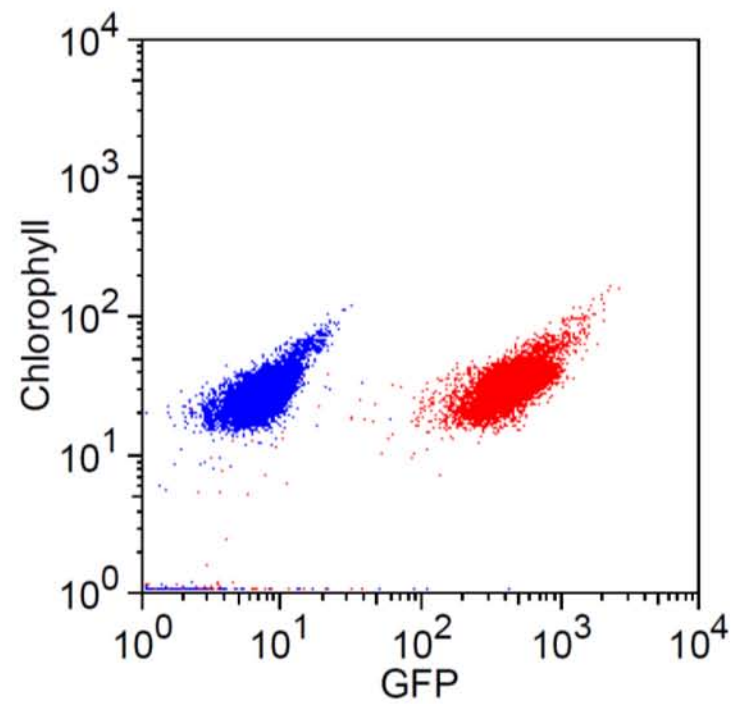

Supplement: Supplemental Information 2 — Green fluorescence (X-axis) is plotted versus autofluorescence of chlorophyll (Y-axis). Data of each population (1,00,000 counts) of transformed cell lines are shown in red for transformed cell lines and in blue for wild type cell line. [file peerj-04-2344-s002.pdf]

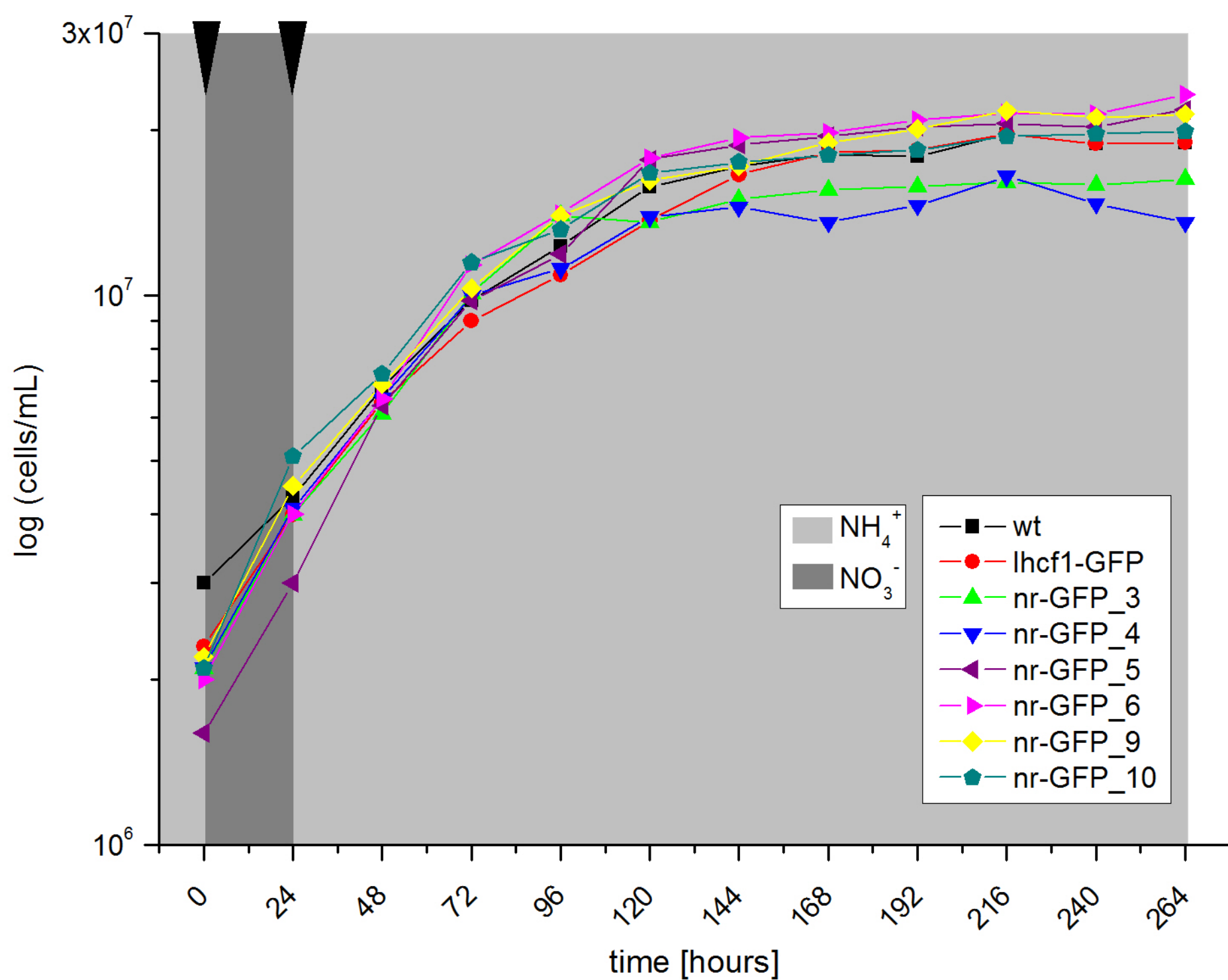

Supplement: Supplemental Information 3 — All cell lines were kept in NH4+-medium (light grey) before transferred into NO3−-medium (dark grey) for 24 h and subsequent transfer back into NH4+-medium. Arrows indicate washing steps and medium change. [file peerj-04-2344-s003.pdf]

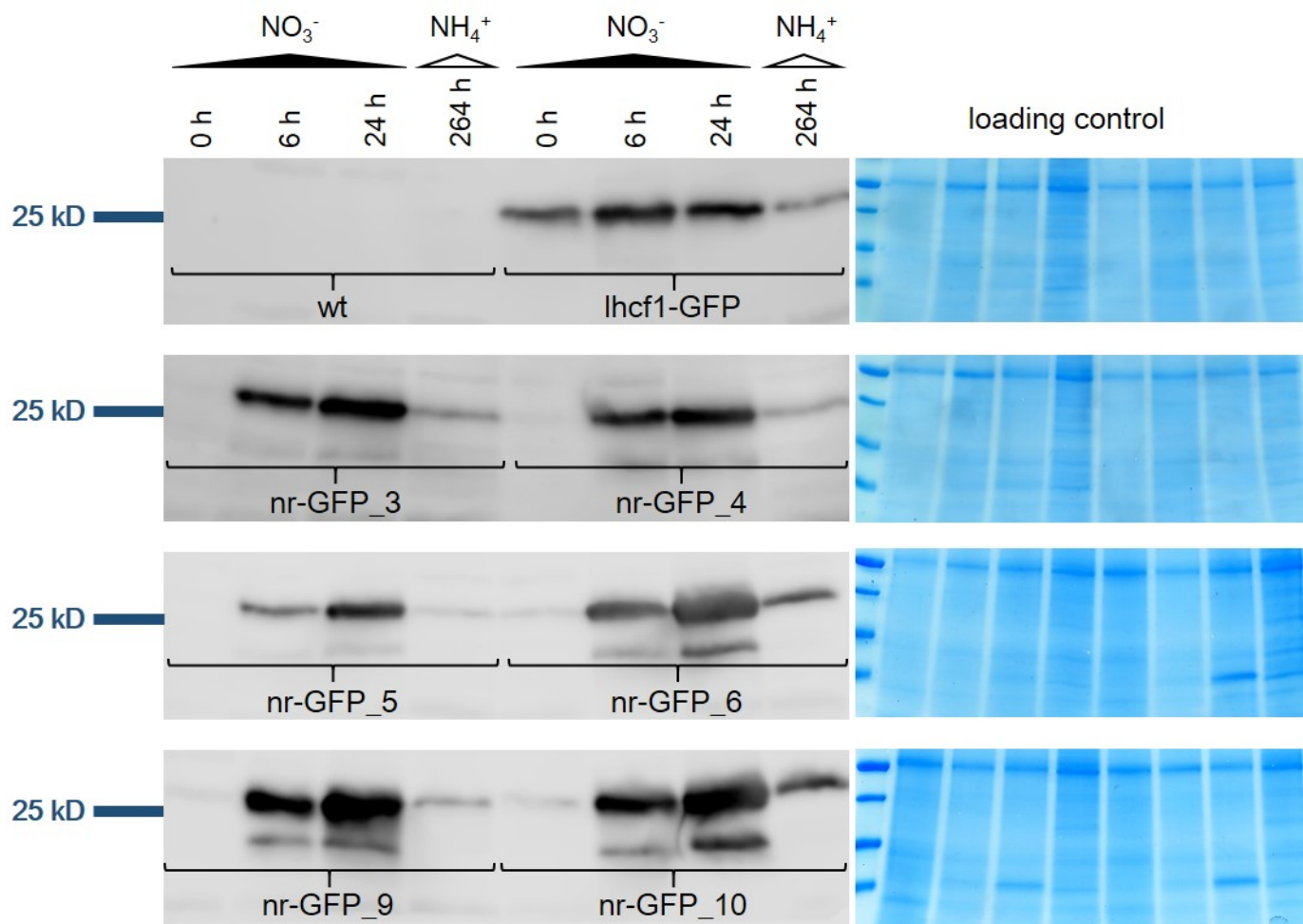

Supplement: Supplemental Information 4 — Total protein extract (3 μg) of P. tricornutum wild type (wt) and transformant cell lines (lhcf1-GFP; nr-GFP_3, _4, _5, _6, _9, _10) and GFP-antibody were used for detection. Left: western blot; right: loading control. GFP-expression by nr promoter is stronger compared to the lhcf1 promoter. GFP (27 kDa) even appears in the coomassie stained loading control of nr-GFP_6, _9 and _10. [file peerj-04-2344-s004.pdf]

GFP

Chlorophyll

Merge

Merge + DIC

0 h

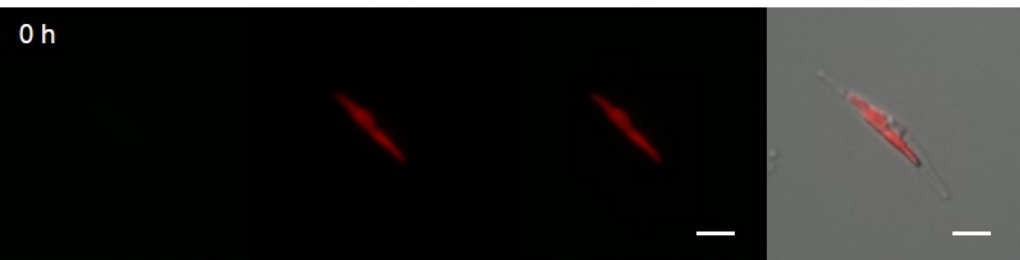

6 h

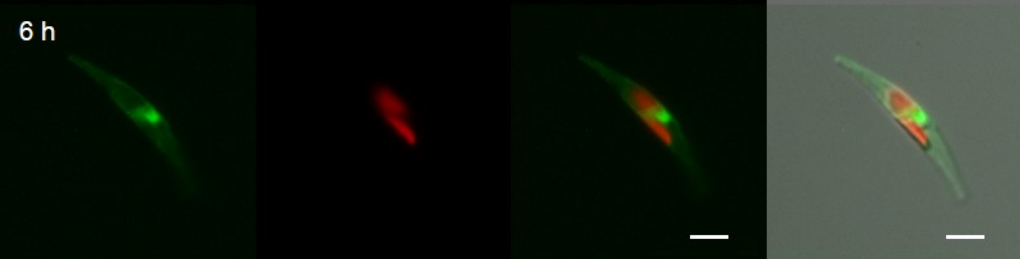

24 h

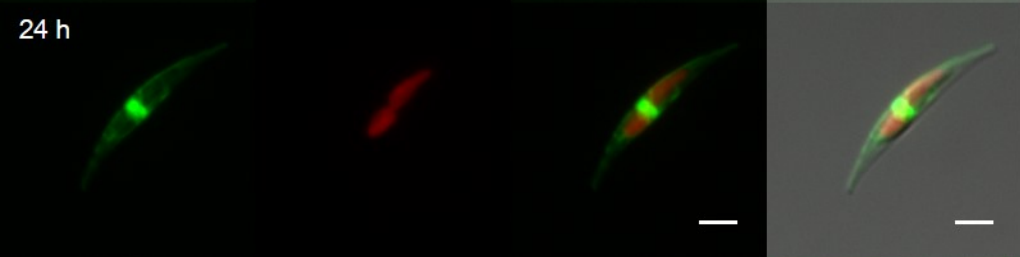

264 h

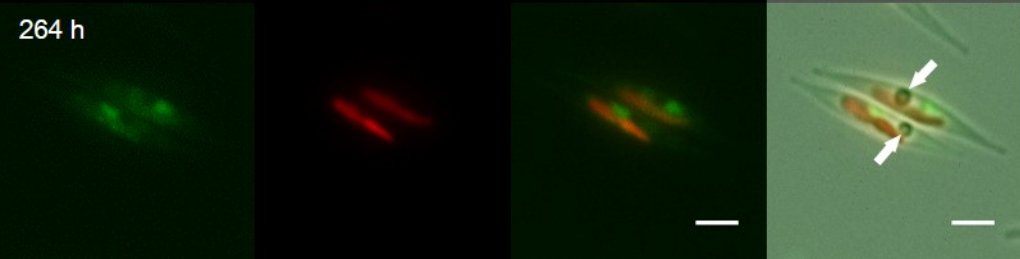

Supplement: Supplemental Information 5 — Images were taken 0 hours (0 h), 6 hours (6 h), 24 hours (24 h) after transfer from NH4+-medium into NO3−-medium, and after back-transfer into NH4+-medium (264 h). Arrows indicate lipid droplets. GFP fluorescence is shown in green, autofluorescence of chlorophyll in red, and Nomarski differential interference contrast (DIC) in grey scale. Scale bars: 5 μm. [file peerj-04-2344-s005.pdf]

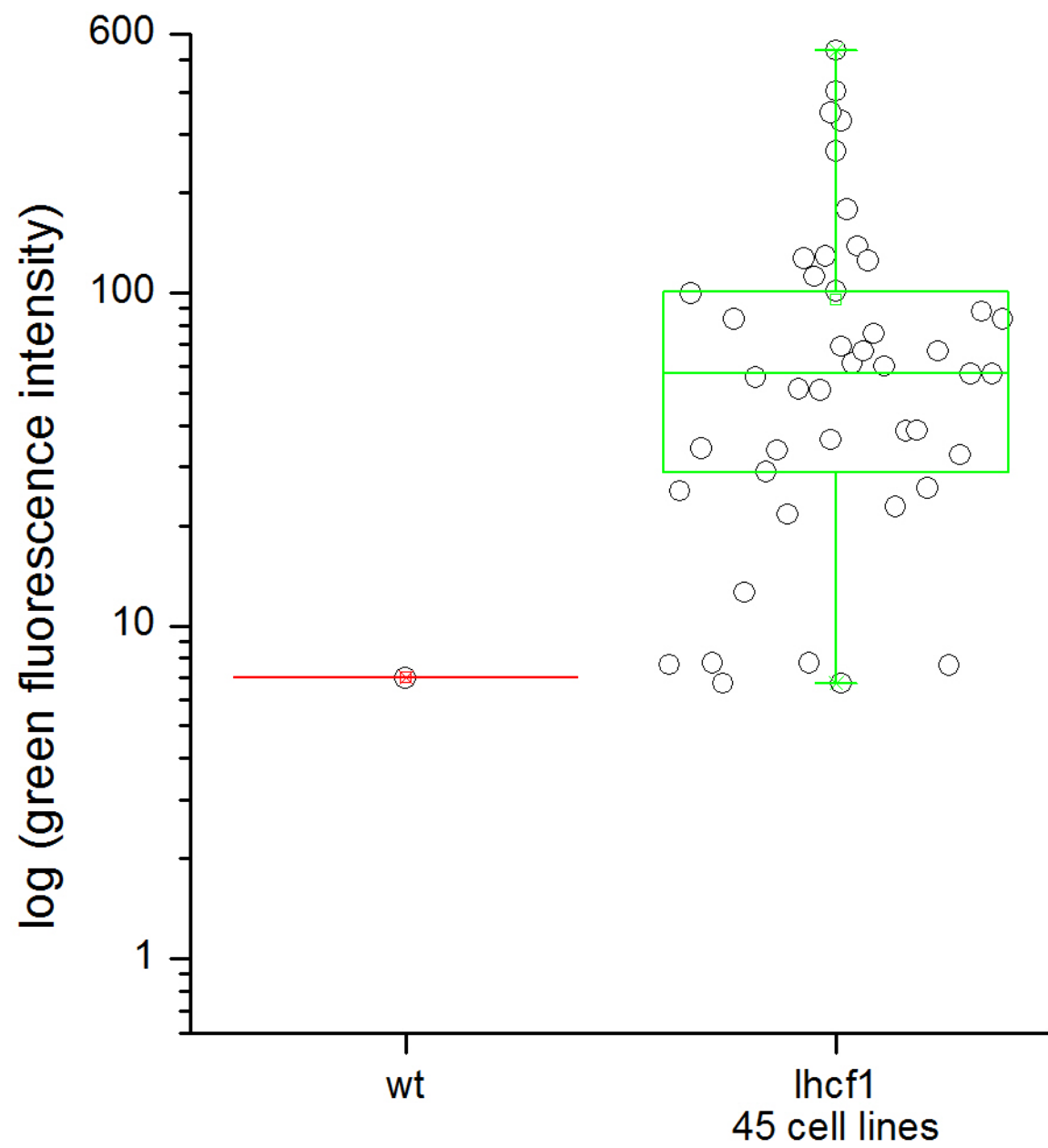

Supplement: Supplemental Information 6 — The intensities were determined for a P. tricornutum wild type cell line (red) and 45 lhcf1-GFP transformed cell lines (green). Whiskers represent min-max-range. [file peerj-04-2344-s006.pdf]
