# Supplementary material for: Rapid induction of GFP expression by the nitrate reductase promoter in the diatom Phaeodactylum tricornutum
Source: PeerJ. 2016 Aug 25;4:e2344. doi: 10.7717/peerj.2344 (PMC5012323; doi:10.7717/peerj.2344)
Supplement: Supplemental Information 7 — Given data show ungated 25% quartile (25%), Median, 75% quartile (75%) and interquartile range (IQR) of the green fluorescence intensities determined for each population (1,00,000 counts). A = medium A (nitrate); B = medium B (ammonium); h = hours. [file peerj-04-2344-s007.docx]

| A) Green fluorescence | | | | | | | | | | | | |
| --- | --- | --- | --- | --- | --- | --- | --- | --- | --- | --- | --- | --- |
|  | 0 h | | | | 3 h | | | | 6 h | | | |
| Sample | 25% | **Median** | 75% | IQR | 25% | **Median** | 75% | IQR | 25% | **Median** | 75% | IQR |
| wt_A | 5 | **6** | 8 | 3 | 5 | **6** | 8 | 3 | 5 | **7** | 8 | 3 |
| lhcf1-GFP_A | 157 | **199** | 250 | 93 | 155 | **197** | 247 | 92 | 143 | **183** | 233 | 90 |
| nr-GFP_3_A | 7 | **9** | 11 | 4 | 7 | **9** | 11 | 4 | 31 | **43** | 56 | 24 |
| nr-GFP_4_A | 6 | **8** | 10 | 4 | 8 | **10** | 13 | 5 | 44 | **56** | 70 | 25 |
| nr-GFP_5_A | 7 | **9** | 11 | 4 | 7 | **9** | 11 | 4 | 47 | **61** | 75 | 28 |
| nr-GFP_6_A | 11 | **14** | 17 | 6 | 13 | **17** | 22 | 9 | 145 | **184** | 231 | 86 |
| nr-GFP_9_A | 8 | **11** | 13 | 5 | 9 | **12** | 16 | 6 | 128 | **162** | 205 | 77 |
| nr-GFP_10_A | 18 | **23** | 30 | 13 | 19 | **25** | 32 | 13 | 208 | **269** | 331 | 123 |
|  | 9 h | | | | 12 h | | | | 15 h | | | |
| Sample | 25% | **Median** | 75% | IQR | 25% | **Median** | 75% | IQR | 25% | **Median** | 75% | IQR |
| wt_A | 5 | **7** | 9 | 3 | 6 | **7** | 9 | 4 | 5 | **7** | 9 | 3 |
| lhcf1-GFP_A | 141 | **181** | 230 | 89 | 138 | **175** | 225 | 87 | 134 | **170** | 220 | 86 |
| nr-GFP_3_A | 75 | **102** | 138 | 63 | 116 | **161** | 219 | 103 | 179 | **240** | 314 | 135 |
| nr-GFP_4_A | 84 | **106** | 135 | 51 | 122 | **158** | 203 | 81 | 168 | **214** | 270 | 102 |
| nr-GFP_5_A | 113 | **157** | 204 | 91 | 182 | **251** | 321 | 139 | 268 | **356** | 447 | 179 |
| nr-GFP_6_A | 414 | **511** | 633 | 219 | 654 | **847** | 1073 | 419 | 977 | **1253** | 1531 | 554 |
| nr-GFP_9_A | 378 | **461** | 558 | 180 | 577 | **744** | 941 | 364 | 825 | **1073** | 1360 | 535 |
| nr-GFP_10_A | 521 | **688** | 876 | 355 | 809 | **1099** | 1401 | 592 | 1213 | **1579** | 1940 | 727 |
|  | 24 h | | | |  |  |  |  |  |  |  |  |
| Sample | 25% | **Median** | 75% | IQR |  |  |  |  |  |  |  |  |
| wt_A | 6 | **7** | 9 | 3 |  |  |  |  |  |  |  |  |
| lhcf1-GFP_A | 136 | **178** | 226 | 90 |  |  |  |  |  |  |  |  |
| nr-GFP_3_A | 321 | **414** | 534 | 213 |  |  |  |  |  |  |  |  |
| nr-GFP_4_A | 312 | **384** | 468 | 156 |  |  |  |  |  |  |  |  |
| nr-GFP_5_A | 482 | **661** | 861 | 379 |  |  |  |  |  |  |  |  |
| nr-GFP_6_A | 1916 | **2371** | 2833 | 917 |  |  |  |  |  |  |  |  |
| nr-GFP_9_A | 1615 | **1961** | 2354 | 739 |  |  |  |  |  |  |  |  |
| nr-GFP_10_A | 2356 | **3067** | 3826 | 1470 |  |  |  |  |  |  |  |  |
|  |  |  |  |  |  |  |  |  |  |  |  |  |
| B) Green fluorescence | | | | | | | | | | | | |
|  | 27 h | | | | 30h | | | | 33 h | | | |
| Sample | 25% | **Median** | 75% | IQR | 25% | **Median** | 75% | IQR | 25% | **Median** | 75% | IQR |
| wt_B | 5 | **7** | 8 | 3 | 5 | **7** | 8 | 3 | 5 | **7** | 9 | 3 |
| lhcf1-GFP_B | 126 | **166** | 214 | 88 | 126 | **163** | 208 | 82 | 127 | **163** | 209 | 82 |
| nr-GFP_3_B | 340 | **444** | 572 | 232 | 329 | **431** | 564 | 235 | 304 | **401** | 532 | 228 |
| nr-GFP_4_B | 338 | **418** | 511 | 173 | 342 | **428** | 536 | 194 | 315 | **398** | 496 | 181 |
| nr-GFP_5_B | 545 | **722** | 945 | 400 | 536 | **699** | 921 | 385 | 519 | **676** | 899 | 380 |
| nr-GFP_6_B | 2188 | **2700** | 3249 | 1061 | 2155 | **2665** | 3272 | 1117 | 2091 | **2590** | 3255 | 1164 |
| nr-GFP_9_B | 1777 | **2163** | 2552 | 775 | 1700 | **2144** | 2547 | 847 | 1601 | **2094** | 2537 | 936 |
| nr-GFP_10_B | 2703 | **3498** | 4416 | 1713 | 2681 | **3481** | 4471 | 1790 | 2569 | **3301** | 4323 | 1754 |
|  | 36 h | | | | 48 h | | | | 72 h | | | |
| Sample | 25% | **Median** | 75% | IQR | 25% | **Median** | 75% | IQR | 25% | **Median** | 75% | IQR |
| wt_B | 5 | **7** | 8 | 3 | 5 | **6** | 7 | 3 | 4 | **6** | 7 | 3 |
| lhcf1-GFP_B | 132 | **170** | 218 | 86 | 137 | **179** | 226 | 89 | 109 | **147** | 191 | 82 |
| nr-GFP_3_B | 279 | **367** | 491 | 212 | 180 | **231** | 303 | 123 | 84 | **113** | 155 | 71 |
| nr-GFP_4_B | 291 | **366** | 458 | 167 | 182 | **221** | 273 | 91 | 84 | **112** | 145 | 61 |
| nr-GFP_5_B | 492 | **630** | 827 | 335 | 299 | **385** | 471 | 172 | 130 | **168** | 227 | 97 |
| nr-GFP_6_B | 1981 | **2398** | 3046 | 1065 | 1447 | **1679** | 1955 | 508 | 630 | **796** | 1064 | 434 |
| nr-GFP_9_B | 1443 | **1859** | 2346 | 903 | 952 | **1142** | 1421 | 469 | 430 | **577** | 763 | 333 |
| nr-GFP_10_B | 2424 | **3053** | 3992 | 1568 | 1723 | **2054** | 2447 | 724 | 813 | **1077** | 1412 | 599 |
|  | 96 h | | | | 168 h | | | | 264 h | | | |
| Sample | 25% | **Median** | 75% | IQR | 25% | **Median** | 75% | IQR | 25% | **Median** | 75% | IQR |
| wt_B | 5 | **7** | 8 | 3 | 5 | **7** | 9 | 4 | 5 | **7** | 8 | 4 |
| lhcf1-GFP_B | 117 | **159** | 201 | 84 | 48 | **58** | 75 | 27 | 12 | **17** | 22 | 10 |
| nr-GFP_3_B | 61 | **79** | 106 | 45 | 24 | **34** | 45 | 21 | 12 | **15** | 20 | 8 |
| nr-GFP_4_B | 58 | **72** | 91 | 32 | 28 | **41** | 58 | 29 | 14 | **19** | 26 | 12 |
| nr-GFP_5_B | 77 | **98** | 127 | 50 | 24 | **33** | 46 | 22 | 9 | **12** | 15 | 6 |
| nr-GFP_6_B | 440 | **522** | 669 | 229 | 131 | **198** | 299 | 168 | 46 | **59** | 79 | 33 |
| nr-GFP_9_B | 304 | **368** | 493 | 189 | 103 | **157** | 234 | 131 | 33 | **43** | 58 | 26 |
| nr-GFP_10_B | 539 | **691** | 927 | 388 | 188 | **286** | 430 | 242 | 59 | **77** | 109 | 51 |
