# Supplementary material for: Rapid induction of GFP expression by the nitrate reductase promoter in the diatom Phaeodactylum tricornutum
Source: PeerJ. 2016 Aug 25;4:e2344. doi: 10.7717/peerj.2344 (PMC5012323; doi:10.7717/peerj.2344)
Supplement: Supplemental Information 8 — Given data show ungated 25% quartile (25%), Median, 75% quartile (75%) and interquartile range (IQR) of the green fluorescence intensities determined for each population (1,00,000 counts). A = medium A (nitrate); B = medium B (ammonium); h = hours. [file peerj-04-2344-s008.docx]

| A) Red fluorescence | | | | | | | | | | | | |
| --- | --- | --- | --- | --- | --- | --- | --- | --- | --- | --- | --- | --- |
|  | 0 h | | | | 3 h | | | | 6 h | | | |
| Sample | 25% | **Median** | 75% | IQR | 25% | **Median** | 75% | IQR | 25% | **Median** | 75% | IQR |
| wt_A | 18 | **22** | 27 | 10 | 18 | **23** | 28 | 10 | 20 | **25** | 31 | 11 |
| lhcf1-GFP_A | 22 | **28** | 34 | 12 | 22 | **28** | 34 | 12 | 22 | **29** | 36 | 13 |
| nr-GFP_3_A | 20 | **26** | 32 | 12 | 20 | **25** | 32 | 12 | 21 | **27** | 33 | 12 |
| nr-GFP_4_A | 17 | **21** | 26 | 10 | 17 | **21** | 27 | 10 | 18 | **23** | 29 | 11 |
| nr-GFP_5_A | 20 | **25** | 31 | 11 | 20 | **25** | 30 | 11 | 20 | **26** | 32 | 12 |
| nr-GFP_6_A | 20 | **25** | 30 | 11 | 20 | **25** | 31 | 11 | 21 | **27** | 33 | 12 |
| nr-GFP_9_A | 16 | **20** | 24 | 8 | 16 | **20** | 24 | 8 | 16 | **20** | 25 | 9 |
| nr-GFP_10_A | 19 | **24** | 30 | 11 | 19 | **24** | 30 | 11 | 20 | **25** | 31 | 11 |
|  | 9 h | | | | 12 h | | | | 15 h | | | |
| Sample | 25% | **Median** | 75% | IQR | 25% | **Median** | 75% | IQR | 25% | **Median** | 75% | IQR |
| wt_A | 21 | **26** | 32 | 11 | 21 | **27** | 34 | 13 | 21 | **27** | 32 | 11 |
| lhcf1-GFP_A | 23 | **30** | 37 | 14 | 24 | **32** | 39 | 15 | 25 | **32** | 40 | 15 |
| nr-GFP_3_A | 22 | **27** | 33 | 12 | 23 | **29** | 36 | 12 | 24 | **30** | 37 | 12 |
| nr-GFP_4_A | 18 | **23** | 28 | 10 | 20 | **25** | 31 | 12 | 21 | **26** | 33 | 12 |
| nr-GFP_5_A | 21 | **26** | 32 | 12 | 22 | **28** | 34 | 12 | 23 | **29** | 36 | 12 |
| nr-GFP_6_A | 22 | **28** | 33 | 12 | 22 | **28** | 34 | 12 | 23 | **29** | 35 | 12 |
| nr-GFP_9_A | 17 | **21** | 25 | 9 | 17 | **21** | 27 | 10 | 18 | **23** | 29 | 11 |
| nr-GFP_10_A | 20 | **26** | 31 | 11 | 21 | **27** | 33 | 12 | 22 | **28** | 34 | 12 |
|  | 24 h | | | |  |  |  |  |  |  |  |  |
| Sample | 25% | **Median** | 75% | IQR |  |  |  |  |  |  |  |  |
| wt_A | 22 | **28** | 34 | 12 |  |  |  |  |  |  |  |  |
| lhcf1-GFP_A | 25 | **33** | 40 | 14 |  |  |  |  |  |  |  |  |
| nr-GFP_3_A | 25 | **31** | 37 | 13 |  |  |  |  |  |  |  |  |
| nr-GFP_4_A | 23 | **30** | 36 | 13 |  |  |  |  |  |  |  |  |
| nr-GFP_5_A | 24 | **31** | 37 | 12 |  |  |  |  |  |  |  |  |
| nr-GFP_6_A | 23 | **29** | 35 | 12 |  |  |  |  |  |  |  |  |
| nr-GFP_9_A | 21 | **26** | 32 | 11 |  |  |  |  |  |  |  |  |
| nr-GFP_10_A | 23 | **29** | 35 | 12 |  |  |  |  |  |  |  |  |
|  |  |  |  |  |  |  |  |  |  |  |  |  |
| B) Red fluorescence | | | | | | | | | | | | |
|  | 27 h | | | | 30 h | | | | 33 h | | | |
| Sample | 25% | **Median** | 75% | IQR | 25% | **Median** | 75% | IQR | 25% | **Median** | 75% | IQR |
| wt_B | 20 | **25** | 31 | 11 | 20 | **25** | 31 | 11 | 20 | **25** | 31 | 10 |
| lhcf1-GFP_B | 23 | **30** | 37 | 13 | 24 | **30** | 37 | 14 | 24 | **31** | 37 | 13 |
| nr-GFP_3_B | 22 | **29** | 35 | 13 | 22 | **28** | 34 | 12 | 22 | **28** | 34 | 12 |
| nr-GFP_4_B | 21 | **27** | 34 | 13 | 22 | **28** | 36 | 14 | 22 | **27** | 34 | 13 |
| nr-GFP_5_B | 22 | **28** | 33 | 11 | 21 | **27** | 32 | 11 | 22 | **28** | 33 | 11 |
| nr-GFP_6_B | 21 | **27** | 33 | 12 | 21 | **27** | 32 | 11 | 22 | **27** | 33 | 11 |
| nr-GFP_9_B | 20 | **25** | 30 | 11 | 19 | **25** | 30 | 10 | 20 | **25** | 30 | 10 |
| nr-GFP_10_B | 21 | **27** | 33 | 12 | 21 | **27** | 33 | 12 | 22 | **27** | 33 | 11 |
|  | 36 h | | | | 48 h | | | | 72 h | | | |
| Sample | 25% | **Median** | 75% | IQR | 25% | **Median** | 75% | IQR | 25% | **Median** | 75% | IQR |
| wt_B | 21 | **26** | 31 | 10 | 19 | **24** | 28 | 9 | 18 | **23** | 29 | 11 |
| lhcf1-GFP_B | 25 | **32** | 39 | 14 | 24 | **30** | 36 | 13 | 23 | **30** | 37 | 14 |
| nr-GFP_3_B | 23 | **29** | 35 | 12 | 22 | **28** | 33 | 11 | 20 | **26** | 32 | 13 |
| nr-GFP_4_B | 22 | **28** | 34 | 12 | 21 | **26** | 31 | 11 | 20 | **25** | 31 | 11 |
| nr-GFP_5_B | 23 | **29** | 34 | 11 | 21 | **26** | 31 | 10 | 21 | **28** | 33 | 12 |
| nr-GFP_6_B | 23 | **28** | 33 | 11 | 21 | **26** | 31 | 10 | 18 | **24** | 29 | 10 |
| nr-GFP_9_B | 20 | **24** | 30 | 10 | 18 | **23** | 27 | 9 | 16 | **22** | 27 | 10 |
| nr-GFP_10_B | 23 | **28** | 34 | 11 | 21 | **26** | 30 | 10 | 19 | **25** | 30 | 11 |
|  | 96 h | | | | 168 h | | | | 264 h | | | |
| Sample | 25% | **Median** | 75% | IQR | 25% | **Median** | 75% | IQR | 25% | **Median** | 75% | IQR |
| wt_B | 22 | **28** | 35 | 13 | 18 | **21** | 28 | 10 | 16 | **19** | 24 | 8 |
| lhcf1-GFP_B | 28 | **36** | 43 | 15 | 20 | **25** | 32 | 12 | 16 | **21** | 26 | 10 |
| nr-GFP_3_B | 23 | **30** | 37 | 13 | 19 | **23** | 29 | 10 | 16 | **19** | 24 | 8 |
| nr-GFP_4_B | 22 | **29** | 35 | 13 | 19 | **24** | 32 | 13 | 16 | **19** | 26 | 11 |
| nr-GFP_5_B | 23 | **29** | 36 | 13 | 17 | **20** | 26 | 9 | 13 | **17** | 21 | 7 |
| nr-GFP_6_B | 21 | **27** | 33 | 12 | 17 | **20** | 25 | 8 | 14 | **17** | 20 | 6 |
| nr-GFP_9_B | 18 | **23** | 29 | 11 | 16 | **19** | 26 | 10 | 13 | **16** | 19 | 6 |
| nr-GFP_10_B | 22 | **29** | 35 | 13 | 18 | **22** | 30 | 11 | 15 | **18** | 23 | 8 |
