# Supplementary material for: Rapid induction of GFP expression by the nitrate reductase promoter in the diatom Phaeodactylum tricornutum
Source: PeerJ. 2016 Aug 25;4:e2344. doi: 10.7717/peerj.2344 (PMC5012323; doi:10.7717/peerj.2344)
Supplement: Supplemental Information 9 — The cells were kept in NH4+-medium for 2 weeks before inoculation into fresh NH4+-medium. After 3 days cells were transferred into NO3−-medium for 24 h and subsequent back-transfer into NH4+-medium and cultivation for another 10 days (264 h). Cell were removed by filtration prior to pH-measurements. [file peerj-04-2344-s009.docx]

| Sample description | pH |
| --- | --- |
| NH_4_^+^-medium without cells | 8,18 |
|  | 8,13 |
|  | 8,16 |
| NH_4_^+^-medium, 3 days cell cultivation | 8,35 |
|  | 8,34 |
|  | 8,33 |
| NO_3_^-^-medium without cells | 8,19 |
|  | 8,22 |
|  | 8,21 |
| NO_3_^-^-medium, freshly transferred cells (1 x washing) | 8,14 |
|  | 8,2 |
|  | 8,18 |
| NO_3_^-^-medium, 24 hours cell cultivation | 8,35 |
|  | 8,78 |
|  | 8,83 |
| NH_4_^+^-medium, freshly transferred cells (1 x washing) | 8,12 |
|  | 8,1 |
|  | 8,12 |
| NH_4_^+^-medium, 264 hours cell cultivation | 7,73 |
|  | 7,77 |
|  | 7,76 |
