# Supplementary material for: Rapid induction of GFP expression by the nitrate reductase promoter in the diatom Phaeodactylum tricornutum
Source: PeerJ. 2016 Aug 25;4:e2344. doi: 10.7717/peerj.2344 (PMC5012323; doi:10.7717/peerj.2344)
Supplement: Supplemental Information 10 — Wild type cells were cultured in triplicates and washed in the same procedure as the one used for the GFP fluorescence measurements. After washing, cells were resuspended in NH4+ or NO3− media at a cell density of 2 × 10^6^cells/ml. PAM measurements were performed in Plastibrand (BRAND GmbH & Co. KG, Wertheim Germany) PMMA cuvettes with an AquaPen AP-C 100 (Photon Systems Instruments, spol. s r.o., Brno, Czech Republic) using the NPQ 2 protocol with actinic light at 700 μmol photons m−1 s−1 and saturating flashes at 2,100 μmol photons m−1 s−1, the blue measuring light was adjusted to 0.0099 μmol photons m−1 s−1. [file peerj-04-2344-s010.docx]

| Time | Transfer of medium | Fv/Fm | | NPQ | |
| --- | --- | --- | --- | --- | --- |
| 0 h | NH_4_^+^ 🡪 NH_4_^+^ | 0.565 | ±0.010 | 0.346 | ±0.021 |
|  | NH_4_^+^ 🡪 NO_3_^-^ | 0.603 | ±0.017 | 0.351 | ±0.019 |
| 24 h | NO_3_^-^ 🡪 NO_3_^-^ | 0.623 | ±0.001 | 0.512 | ±0.093 |
|  | NO_3_^-^ 🡪 NH_4_^+^ | 0.585 | ±0.006 | 0.477 | ±0.036 |
| 264 h | NH_4_^+^ 🡪 NH_4_^+^ | 0.284 | ±0.004 | 2.305 | ±0.115 |
